# Metabolic Lateralization in the Hypothalamus of Male Rats Related to Reproductive and Satiety States

**DOI:** 10.1007/s43032-019-00131-3

**Published:** 2020-01-06

**Authors:** David S. Kiss, Istvan Toth, Gergely Jocsak, Tibor Bartha, Laszlo V. Frenyo, Zoltan Barany, Tamas L. Horvath, Attila Zsarnovszky

**Affiliations:** 1 Department of Physiology and Biochemistry, University of Veterinary Medicine, Istvan u. 2, Budapest, 1078 Hungary; 2 Department of Animal Physiology and Animal Health, Faculty of Agricultural and Environmental Sciences, Szent Istvan University, Pater Karoly u. 1, Godollo, 2100 Hungary; 3 Department of Comparative Medicine, Yale University School of Medicine, 310 Cedar Street, New Haven, CT 06520-8016 USA

**Keywords:** Hypothalamic asymmetry, Mitochondrial metabolism, Melanocortin system, Regulation of homeostasis, Gonadectomy, Feeding state

## Abstract

The hypothalamus is the main regulatory center of many homeostatic processes, such as reproduction, food intake, and sleep-wake behavior. Recent findings show that there is a strongly interdependent side-linked localization of hypothalamic functions between the left and right hemispheres. The goal of the present study was to trace functional asymmetry of the hypothalamus related to the regulation of food intake and reproduction, in male rodents. Subjects were examined through measurements of mitochondrial metabolism ex vivo. Impact of gonadectomy and scheduled feeding was tested on the modulation of hypothalamic metabolic asymmetry. Results show that in male rats, functional lateralization of the hypothalamus can be attributed to the satiety state rather than to reproductive control. Fasting caused left-sided metabolic dominance, while satiety was linked to the right hemisphere; trends and direction in sided dominance gradually followed the changes in satiety state. Our findings revealed satiety state-dependent metabolic differences between the two hypothalamic hemispheres. It is therefore concluded that, at least in male rats, the hypothalamic hemispheres control the satiety state-related functions in an asymmetric manner.

## Introduction

The hypothalamus is the main regulatory center of many homeostatic processes, such as reproduction, food intake, and sleep-wake behavior. As a result of three decades of thorough investigations, an appreciable knowledge has already been collected on the particular function of different hypothalamic nuclei, and the unilateral, complex interaction between them. However, with few exceptions, most studies neglect an indispensable aspect of the hypothalamus: a possible interplay between the two hypothalamic sides leading to a potential functional asymmetry.

It is well-established that cerebral functions, as a beneficial outcome of evolutionary processes providing an efficient use of brain resources, show different degree of lateralization. Functional asymmetry has also been detected in other brain areas such as the hippocampus, habenulae, and thalamus [1–3]. Although data indicating that the two sides of the neuroendocrine hypothalamus are specialized to the regulation of distinct functions have already been obtained earlier, hypothalamic asymmetry per se has been neglected for a long time. In the late 1970s and early 1980s, Gerendai et al. [4] and other research groups [5, 6] described several aspects of the asymmetrical regulation of the reproductive processes providing basic data on both male and female hypothalamic-pituitary-gonadal axis in different species.

Recently, several research groups, including ours, have provided evidence to support those early observations*.* In line with the findings of Cruz et al. and López et al. [7, 8], we found that a predetermined functional sidedness exists in the hypothalamic regulation of female reproduction, and this lateralization shows a strongly estrogen-dependent right-sided dominance [9, 10]. Besides this, we proved that food intake-related hypothalamic functions are also lateralized in female rats [10].

Hypothalamic lateralization is, however, not restricted to females. Pronounced morphological differences in the central nervous system-testis axis of male rodents, and humans provide plausible hypotheses for lateralized function at the hypothalamic level [11–14]. In fact, gonadotropin-releasing hormone (GnRH) cells were found in higher numbers in the right side of the brain [15] that is consistent with the larger amount of the neurohormone produced in the right side, detectable however exclusively in the morning period [16]. Moreover, the relation between gonads and the hypothalamic regulatory center proved to be lateralized, too, since the GnRH cells were more responsive to the removal of the right testis compared to the removal of the left [17].

Besides the regulation of reproduction, the hypothalamus controls a number of other physiological processes as well. Although the regulation of these functions is based on the action of distinct pathways and neural circuits, there is an extended overlap and strong interplay between the relevant neuron populations and hormones [18–20]. For example, gonadal steroids, estrogen and testosterone, do not exclusively coordinate the reproductive physiology but also affect several aspects of the regulation of appetite and energy expenditure [21, 22]. Depending on the nutritional state, the melanocortin system adjusts glucose homeostasis, fatty acid metabolism, and the satiety state itself according to the actual needs [23]. Beyond the metabolic inputs, the melanocortin system receives first-order neural projections from the hypothalamic master clock located in the suprachiasmatic nucleus (SCN) to align behavioral (such as food intake) and biochemical processes with the day/night cycle during normal conditions. This bipolar input system ensures that energy metabolism is coordinated in an optimal temporal pattern (reviewed by Huang et al. [24]).

Thus, the consideration of the lateralized control of reproductive events is now recommended and should be taken into account regarding the control of feeding behavior and food intake [25, 26].

In this work, we further investigate this suggestion in a number of different experimental conditions. We propose that hypothalamus-controlled regulatory processes are lateralized in male rats similarly to that found in females. This functional asymmetry is most likely manifested in the regulation of the two most fundamental activities in all animals: food intake and reproduction [27]. Beyond this, we also reveal how the circadian rhythm and the control of metabolism are connected in the frames of hypothalamic functional asymmetry.

The hypothalamic regulation of reproduction, food intake, and circadian control requires high energy supply that is covered by mitochondrial ATP production [28]. Mitochondrial activity is fine-regulated and steadily adjusted to the actual energy needs; therefore, the mitochondrial respiration level precisely denotes the actual cellular energy consumption [29, 30]. Based on this, analyzing mitochondrial bioenergetics provides a well-suited method to reveal the activity of hypothalamic regions and circuits that are involved in the response to experimental (or naturally occurring) cues.

## Materials and Methods

### Animals

Intact and gonadectomized Wistar rats (*Rattus norvegicus*, breed: Crl:[WI]BR) were used to examine the effects of satiety states and presence or absence of male gonadal steroids. Studies were conducted at the University of Veterinary Medicine (Budapest, Hungary) in accordance with the Directive 2010/63/EU and was approved by the Animal Health and Animal Welfare Directorate of the National Food Chain Safety Office (permit no.: XIV-I-001/2202–4/2012 and PEI/001/665–8/2015).

Animals were obtained at least 5 weeks before the experiments (vendor: Semmelweis University, Basic Medical Science Center; Budapest, Hungary) and were kept on regular rat chow (vendor: FarmerMix Kft., Zsambek, Hungary) and ad libitum tap water.

### Treatment of Animals and Experimental Setups

#### Examination of Hypothalamic Lateralization Depending on Presence of Testes and Food Deprivation (Experiment A)

About 3 weeks before the experiments, 7-week-old animals were sorted into two groups (seven animals/groups), both kept in controlled 12-h-long dark and light cycles. One group remained intact, while the individuals in the other were gonadectomized under standard anesthesia of ketamine + xylazine combination (75 mg/kg ketamine, 0.2 mg/animal xylazine, subcutaneously). Here, the testes were removed through a single incision of the scrotum. After closing the wound, butorphanol (2.0 mg/kg) was administered for postoperative analgesia together with subcutaneous saline infusion. After gonadectomy, and in the experimental period, the animals were kept in groups of two. Before the experiment, the animals were randomly separated into two groups; one of them remained ad libitum fed, while the other was fasted for 24 h before sacrifice (quick guillotine decapitation in deep isoflurane narcosis right at the end of the dark period) with constant water supply. In summary, the experimental groups used are intact ad libitum fed males (T + ad lib.), intact fasted fed males (T + fasted), castrated ad libitum fed males (cast. + ad lib.), and castrated fasted fed males (cast. + fasted).

#### Examination of Satiety State-Dependent Lateralization under Scheduled Feeding in Male Rats (Day Time Feeding, Experiment B)

After acclimatization (see above), 11 groups (6 animals/groups) of 6-week-old intact male animals were set for programed feeding for 4 weeks. Chow availability was limited to a 1-hour period right after the dark period (portion was set for 12–15 g/animal depending on age). Animals were sacrificed at 11 particular time points of the feeding cycle (6, 2, 1 h and right before onset of feeding, right after and 1, 2, 3, 5, 7, 9 h after the 1-h feeding period).

#### Examination of Satiety State-Dependent Lateralization under Scheduled Feeding (Night Time Feeding, Experiment C)

After acclimatization (see above), 3 groups (7 animals/groups) of 6-week-old intact male animals were set for programed feeding for 4 weeks. They were fed with restricted chow portion (see above) in the middle of their night (active) period, timed 5 h after the light switch-off. Animals were sacrificed at three particular time points of the feeding cycle: 1 h before feeding time, right at feeding time, and 1 h after feeding time.

#### Examination of Circadian Rhythm-Dependent Lateralization under Ad Libitum Feeding (Experiment D)

After acclimatization, 8 groups (6 animals/groups) of 6-week-old intact male animals were set for programmed day-night cycle with ad libitum feeding all over the day, for 4 weeks. Animals were sacrificed at 8 particular time points of the day-night cycle around the phase switches (at both phase switches and 1, 2 h before and thereafter).

### Preparation of Mitochondria Fractions and Measurement of Oxygen Consumption

Mitochondrial fractions (containing both perikaryal and synaptosomal mitochondria) were obtained from the separated left and right hypothalamic sides (also termed as hemispheres), and then mitochondrial oxygen-consumption was measured. The experimental design of isolation and mitochondrial oxygen-consumption measurement (including the necessary reagents and equipment) is detailed by Toth et al. [9] and Kiss et al. [31]

### Data Analysis

Although all five mitochondrial respiration states (as explained by Kiss et al. [31]) were measured and evaluated, only mitochondrial respiration rates (*mrr*) from state 3 (St3) and state 4 (St4) mitochondrial respiration were analyzed in this study. St3 gives a plausible insight into mitochondrial metabolism since ADP/ATP ratio potently regulates mitochondrial activity [32], while St4 indicates the degree of uncoupling and the activity of alternative oxidases, two factors that play an important role in transient down-regulation of ATP biosynthesis when cellular energy needs drop.

The collected data were evaluated from three aspects as follows:

I. Based on *mrr* values, we determined the side of higher metabolic rate termed as left- or right-sided dominance (i.e., indicating the direction of sidedness). Because of the internal error of the applied protocol, we set up a strict requirement, and sidedness as a term was only used if the difference between left and right hypothalamic sides of the individual was 20% or higher [31].
II. Percentage of sidedness (i.e., 20% or higher) expressed the degree of the asymmetry (i.e., fold difference between the activity of the two sides).
III. Changes of separate left and right side *mrr* data were plotted in the form of mean values against time to trace individual pattern of the left and right side values on interval scales (see time-scheduled experiments).

Fisher’s exact test was applied to evaluate sidedness and two-way ANOVA with Bonferroni posttests to compare degree of asymmetry between groups by Prism 5 (GraphPad Software Inc., San Diego, CA).

## Results

Results are partitioned according to the experimental setups listed in the *Materials and Methods.*

### Examination of Hypothalamic Lateralization Depending on Presence of the Testes and Food Deprivation

First, we determined the percentage of animals with metabolic sidedness of the hypothalamus in each group, as approached in earlier studies [10, 31]. In St3, there was no change in the proportion of sided vs. not sided animals in most the groups (around 70%), except for castrated + fasted animals, where only 50% of the individuals were metabolically imbalanced.

**Fig. 1:**
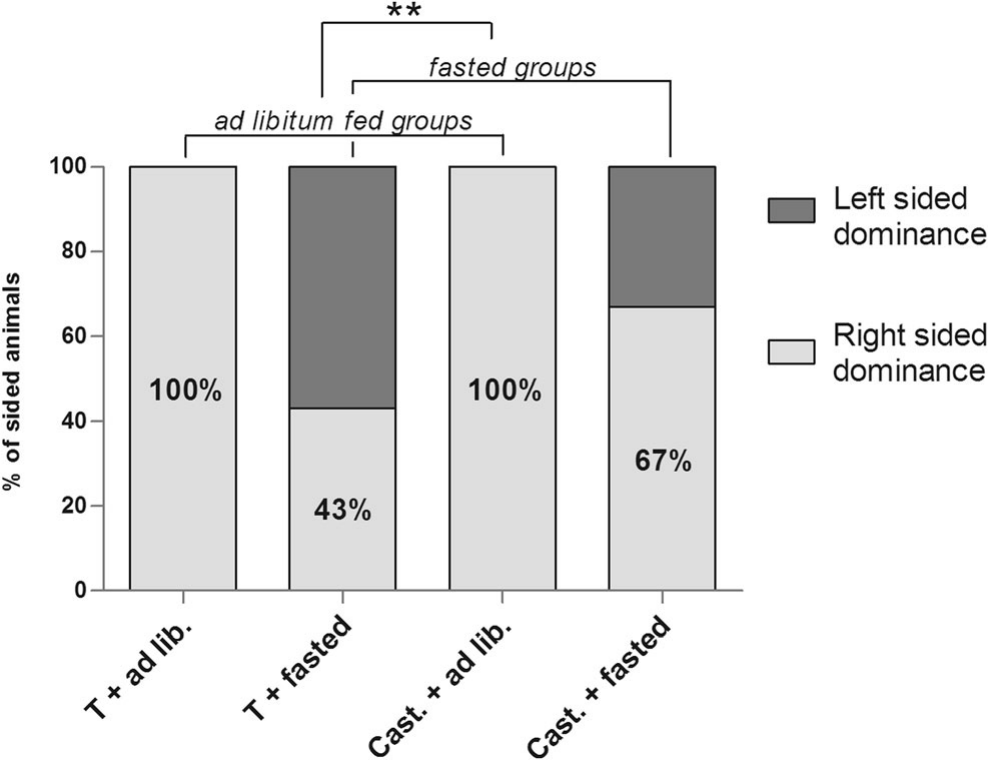
Share of left and right side dominance in male rats in respiratory state 3. Animals in ad libitum fed groups showed right sided dominance, while in fasted animals left-sided dominance could also develop in approximately 50% of the examined individuals (*p* = 0.0058)

In order to elucidate the share of left and right side in hypothalamic sidedness, sided animals were taken into account, according to the above-described. In all ad libitum animals, the higher metabolic rate was detected exclusively on the right hypothalamic side, regardless of castration. In contrast, in fasted animals, the metabolic activity of the sides was nearly balanced, as only around 40–60% of the individuals were right-sided (Fig. 1).

**Fig. 2:**
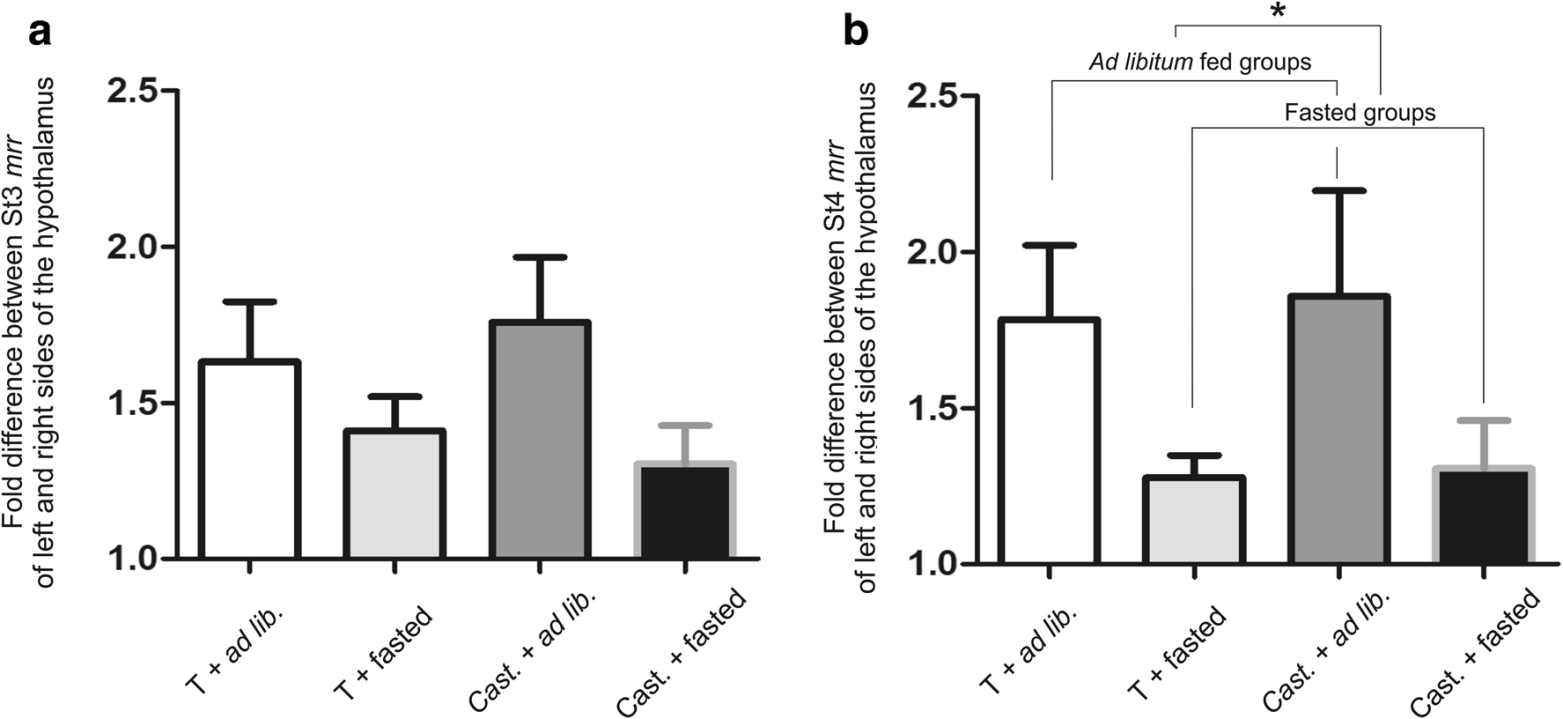
Degree of hypothalamic asymmetry in St3 and St4 in male rats. Sidedness was remarkably higher in ad libitum fed animals in both St3 and St4 (*p* = 0.0384.)

We also analyzed the degree of metabolic lateralization by the fold differences in mitochondrial metabolism between the left and right hypothalamic sides (St3 and St4) (Fig. 2). Our results show that sidedness was remarkably higher in ad libitum fed animals, and it even reached a significant level in St4 (*p* = 0.0384). In contrast, orchiectomy, regardless of the satiety state, led to only slight alteration of metabolic differences between the two sides. It has to be noted that in ad libitum groups, regardless of gonadectomy, there were a few animals showing balanced metabolic phenotype (i.e., L-R difference is around 1) in St3 and St4, as well.

### Examination of Satiety State-Dependent Lateralization under Scheduled Feeding in Male Rats (Day Time Feeding)

**Fig. 3:**
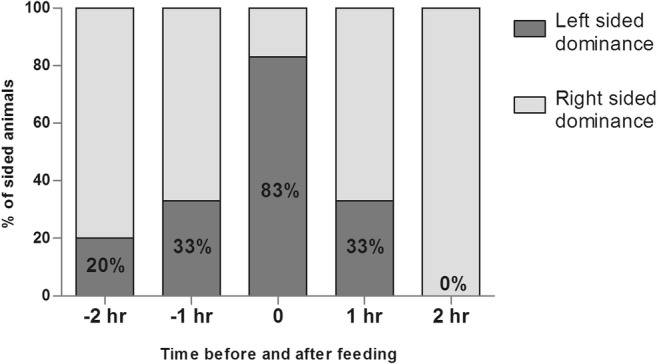
Share of left and right side dominance (St3) in the hypothalamus of scheduled fed male rats around onset of alimentation.

For further clarification of the metabolic phenomena related to feeding behavior in the hypothalamus, we performed scheduled feeding on intact male rats and examined the intensity of hemispheric metabolism before and after feed intake. Animals in all sampling groups (time points before and after feeding) showed a sidedness of around 20%; thus the degree of asymmetry was not notably changed with the satiety state. However, sidedness showed a continuously and exponentially increasing left-sided dominance after 21 h of fasting; meanwhile, access to chow re-established the right-sided dominance with the same tendency after 2 h (1 h on Fig. 3.).

**Fig. 4:**
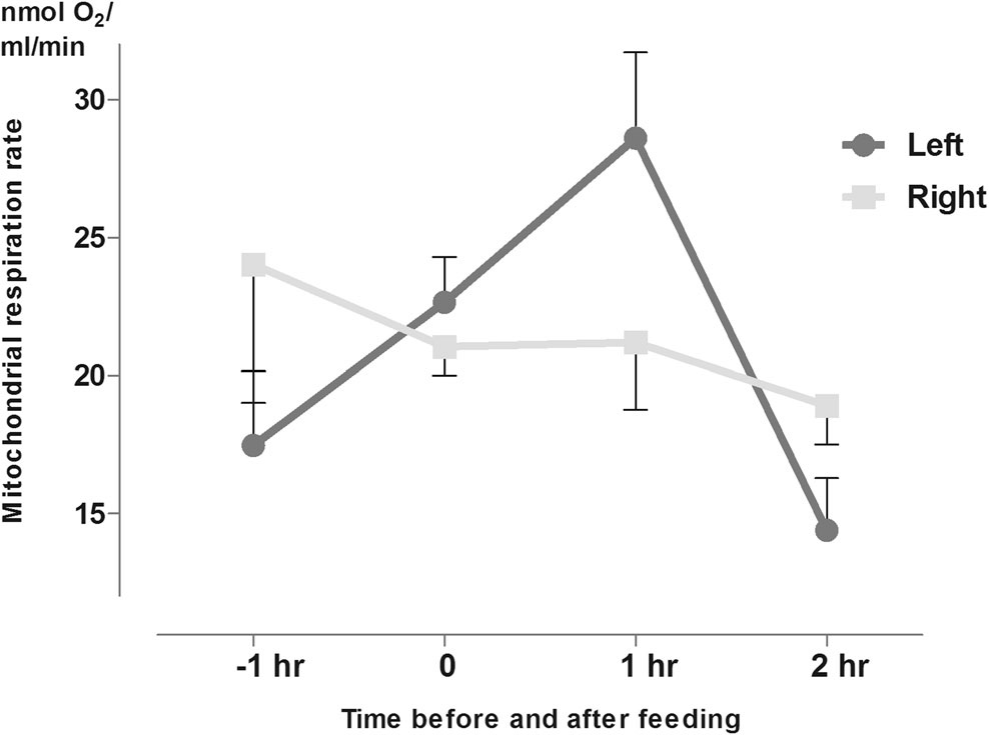
Metabolic activity pattern in St3 of the left and right hypothalamic hemispheres around the onset of alimentation in male rats scheduled-fed in the day-time*.*

Separated left and right side *mrr* mean values strengthened the trend of the increasing left-sided dominance around feeding period; however, the larger deviation of individual values at the end of the feeding period (1 h on Fig. 4.) resulted in a seemingly higher left sidedness followed by rash lowering to pass the dominance to the right side.

### Examination of Satiety State-Dependent Lateralization under Scheduled Feeding in Male Rats (Night Time Feeding)

In experiment B, the onset of feeding period and the night-day transition coincided. This was to follow experimental procedures of earlier investigations to maintain the comparability with them. To avoid the presumptive impact of circadian phase changes on the metabolic status of the two hypothalamic sides, in experiment C, we tested the impact of restricted food availability if a short feeding period was inserted into the animals’ active period (i.e., physiologic feeding time) and well separated from any strong circadian events.

**Fig. 5:**
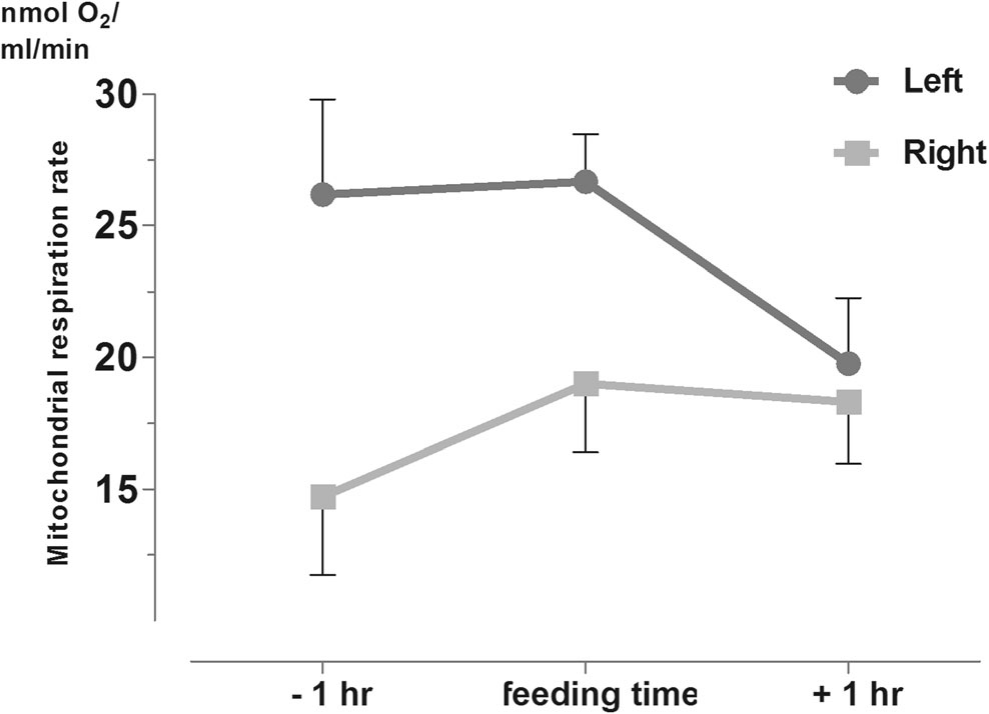
Metabolic activity pattern (St3) of the left and right hypothalamic hemisphere around the onset of alimentation in male rats scheduled fed in the night-time (for left vs. right side values *p* = 0.0039).

Results showed that the left hemisphere was more active as the feeding time approached, compared to the right side. After the onset of feeding, the more active metabolism on the left side shifted to the right side, fully supporting the findings in experiment B. With regard to the degree of asymmetry, fold difference between sides was definitely higher at the last stage of fasting (time point − 1 h) reaching a significant level, whereas after feeding, metabolic activity of the two sides became anatomically balanced (Fig. 5.).

### Examination of Circadian Rhythm-Dependent Lateralization under Ad Libitum Feeding

In order to disclose the impact of circadian phase-induced changes on the metabolic lateralization, here, we tested the effects of the circadian rhythm only, with free access to rat chow.

Unexpectedly, the left hypothalamic side absolutely dominated over the right side throughout the day, as altogether 90% of all animals drawn into this experiment showed left sidedness, regardless of the sampling time. Analyzing the plotted *mrr* data, both the night-to-day and the day-to-night switch resulted in remarkable alterations in the metabolism of the left-right hypothalamic hemispheres. Around the night-to-day switch, metabolic activity of both hemispheres showed a well-detectable (but not significant) increase. Activities peaked when switching on the light, after which returned to the lower values observed during the second phase of the night period. Differences in the intensity of metabolic activity between the two sides were not notable; moreover, in most sampling time points, they were rather balanced.

**Fig. 6:**
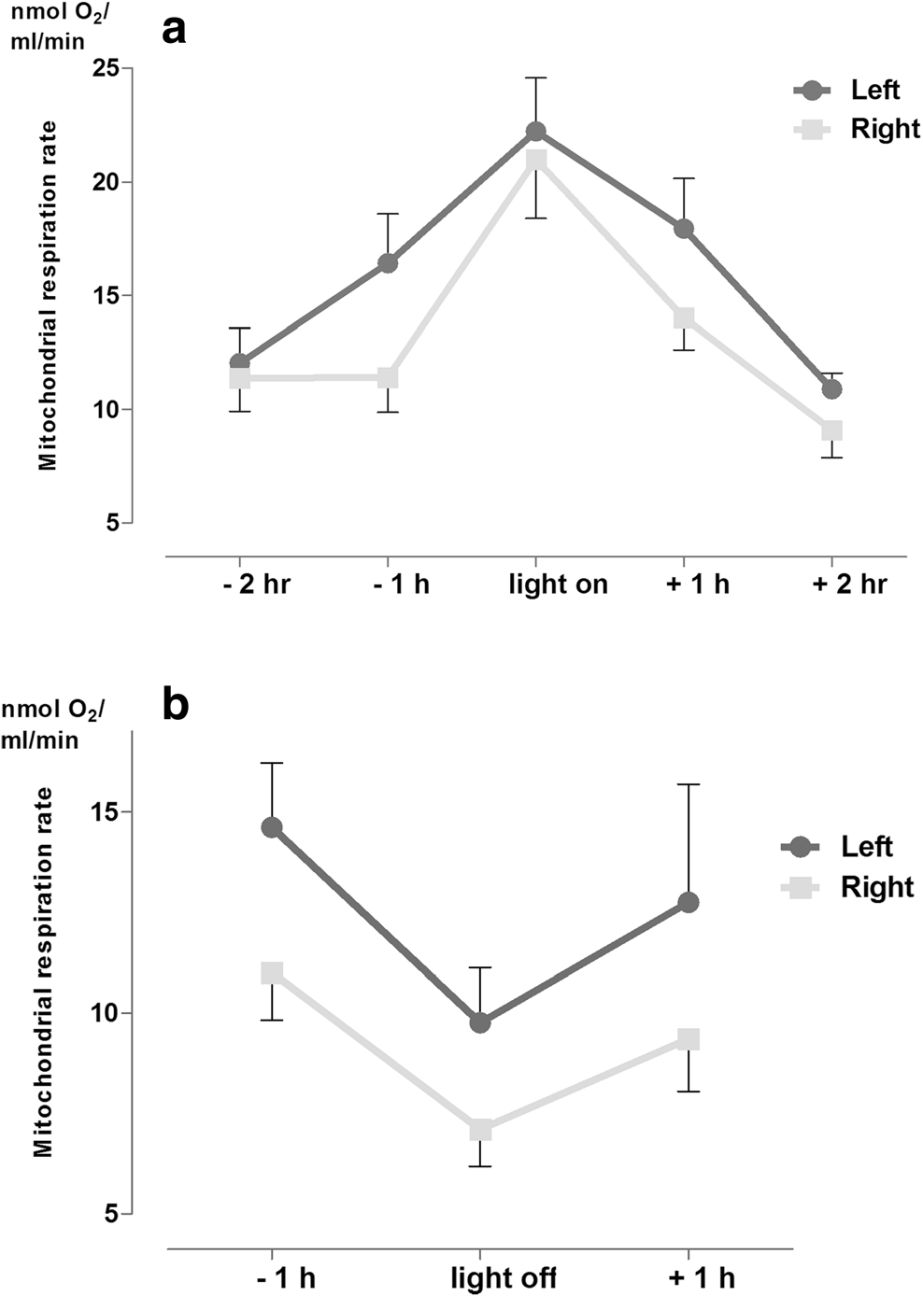
Metabolic activity pattern (St3) of the left and right hypothalamic hemisphere around (A) night-to-day and the (B) day-to-night switches (for left vs. right side in the day-to-night switch group *p* = 0.0081)

Around the day-to-night switch, the intensity of metabolism showed the opposite pattern: the metabolism of both hemispheres converged to a negative peak right at the day-to-night switch. Interestingly, the degree of asymmetry was significantly higher than around the night-to-day switch, regardless of the sampling time (Fig. 6.).

## Discussion

The goal of this study was to observe functional asymmetry in the hypothalamus in male rodents under physiological conditions (intact, ad libitum fed) and under basic but potentially determinant impacts targeting the hypothalamic centers of reproduction, food intake, and circadian control. The applied experimental conditions were chosen and set in order to elicit such hypothalamic (mostly short-term) processes that can be detectable on a metabolic level (i.e., by measuring mitochondrial metabolism). Moreover, our present results yielded the unconcealed possibility of comparing the basics of sided metabolic activity in male and female hypothalamus [10].

### Reproduction

Available data regarding the lateralized regulation of reproduction in male animals are very sporadic and rather inconsistent [4]. One of the pioneering research groups in this field, Bakalkin et al. [15] observed higher GnRH content in the right side of the hypothalamus compared to the left one, in male mice, while Mizunuma et al. [33] found no differences between the two sides. Besides GnRH production, aromatase activity of the preoptic, anterior, and posterior hypothalamic regions was found to be lateralized, with a direction depending on the developmental stage in rodents [34]. A right-sided dominance with regard to the control of reproduction also seemed to be supported by deafferentation studies [16].

Our research group has previously reported the dominance of the right side of the hypothalamus in the regulation of reproduction in female rats [10]. In line with earlier suggestions, we proved that the proportion of (in this respect) sided animals changed significantly depending on the presence or absence of the gonads/gonadal steroids, and changes in metabolic status of the right hypothalamic side follow the plastic changes of the GnRH-circuitry described throughout the estrus cycle [10, 35]. In contrast to this, the present results show that in males, neither bilateral orchiectomy nor food deprivation resulted in notable alteration in the ratio of sided vs. not sided individuals. Share of left- vs. right-sided dominance (i.e., which side of the hypothalamus showed higher metabolism), as well as the degree of lateralization between hypothalamic sides (fold differences between the activity of the two sides of the hypothalamus), also remained independent by male gonad (experiment A).

Altogether, it is suggested that male reproduction, at least in rodents, is either not regulated in a lateralized manner in the hypothalamus or remains hidden because of more intense metabolic events attributed to other functions.

### Food Intake

Function and role of the circuitry regulating food intake and energy expenditure is well-established and known in fine details; however, there is almost no data available yet on how hypothalamic hemispheres share their tasks, if at all, in the control of these functions.

According to the present results, fasting and changes in satiety state strongly determine differences in the orchestration of metabolism between the two hypothalamic hemispheres. In ad libitum fed conditions, within the cohort of sided males, the right hypothalamic hemisphere was metabolically more active, while 23 h after food deprivation, the left side took over the dominance in the intensity of metabolism in about the half of the individuals (experiment A). Analyzing the degree of metabolic asymmetry, this phenomenon became further supported in males since in ad libitum fed animals, higher degree of hypothalamic asymmetry was detected. It is therefore suggested that fasting enhances the metabolism on the left hypothalamic side, abolishing the right-sided dominance detectable under satiety, that led to a balance of the two sides. This is fully in line with earlier findings in female rats, where fasting balanced the proportion of left and right sided dominance as well [10]; nevertheless, in those experiments, metabolic actions in the hypothalamus related to feeding-control were, at least in part, masked by the overwhelming influence of gonadal steroids and thus remained masked by the estrogen-induced events.

Experiments on strictly scheduled-fed animals supported the concept as well and demonstrated that the metabolic switch between the hypothalamic sides occurred gradually while fasting culminated. After feeding, the dominance of the right side returned with the same trend (experiment B).

Since in experiment B, the onset of feeding period coincided with the beginning of the lights-on (i.e., resting) phase of the day-night cycle; in order to confirm that the above data originate exclusively from satiety state, in experiment C, scheduled feeding was separated in time from the day-night switch as much as possible. In case when strong circadian events were out of view, scheduled feeding alone could evoke lateralized changes in the intensity of metabolism between hypothalamic hemispheres. Here, as well as in experiment A, the fasting period was linked to the dominance of the left side, while satiety reversed this to a right-sided dominance. This result fully strengthens the view we obtained in experiment B, where approaching feeding time was gradually paralleled with enhanced left-side activity, leading to the conclusion that forecast of the daily feeding time and/or food-seeking activity is somehow bound to the left hypothalamic hemisphere, while the right side dominates in satiety-related events. This may also mean that besides the ipsilateral interconnection between NPY-AgRP neurons (hunger cells) and POMC neurons (satiety cells) [36], a contralateral interconnection, either anatomical or humoral, between the aforementioned distinct types of neurons may also exist. Further tracing studies are necessary to clarify this question.

In line with these, earlier studies have already described the existence of unilateral feeding pathways connecting hypothalamic structures to brain areas with asymmetric functions [25, 37, 38]. Beyond this, left-sided dominance under food deprivation has been observed in cats and rabbits [25, 39] suggesting that this kind of bias in food searching and food intake-related motivation might be a general phenomenon in many mammals.

These data imply that the well-studied dynamic interactions between orexigenic (stimulating appetite and food intake) and anorexigenic (suppressing appetite and food intake) hypothalamic nuclei might differ on the left and right sides. Although these kind of neuronal activations possibly occur in both hypothalamic sides, our results suggest that the satiety state of the animals primarily depends on the right hypothalamic side, while orexigenic activation of the melanocortin system dominates on the left hypothalamic side (however, to some extent it probably happens in both sides).

According to other earlier studies, satiety and feeding-related reward mechanisms could be bound to hemispheric lateralization with regard to different brain areas not only in the hypothalamus but also in other brain regions. In humans, for example, the right prefrontal cortex appears to play a pivotal role in the control of reward-generating mechanisms, while this has not been proven for the left counterpart. In line with this, human gourmand syndrome (abnormal passion for eating palatable food) is associated to right frontal damage [40–42]. However, whether this functional asymmetry could be attributed to asymmetries in the peripheral autonomic nervous system or to the asymmetric connections between the hypothalamus and cortex, or both [43], remains to be clarified.

### Circadian Rhythm

Most experiments suggesting any kind of asymmetry related to the hypothalamic function were executed relatively close or right on time of circadian day-night switch. To compare our results to those earlier findings, we generally applied the same experimental setups. A lateralized functioning of hypothalamic areas in the control of circadian events has been assumed recently. Followed by several studies, de la Iglesia et al. [44, 45] were the first to observe that the bilaterally localized two parts of the suprachiasmatic nucleus; the principal circadian pacemaker of the body could be functionally split from each other resulting in an asynchronous and biphasic control over the subordinated mechanisms, such as regulation of reproduction.

In experiment D, we tested the effects of the two main circadian phase switches to unravel whether the circadian rhythm exerts any detectable impact on the hypothalamic metabolic lateralization in our other experiments (especially in experiment B). Unexpectedly, the metabolic status of the hypothalamus around transitions between circadian periods (day-to-night and night-to-day, with full time ad libitum feeding) resulted in a left-sided dominance with degree varying throughout the day. This seems to contradict the right-sided dominance experienced in case of the scheduled-feeding experiments (A, B, C). It is noteworthy that the activity of the two sides changed in a similar manner. Around the night-to-day switch, both hemispheres increased their metabolism in an almost parallel manner, while by the day-to-night transition, the opposite happened; the metabolic activity of both sides was lowered.

Results suggest that without entraining effects of restricted food availability, circadian oscillations overwhelmingly dominate the hypothalamic activity establishing cyclically repeated metabolic changes throughout the day. In these cyclic changes, the two hypothalamic hemispheres undergo simultaneously occurring events, however, with different shares. Simultaneous metabolic changes in the two hypothalamic hemispheres related to day-night switches seem to be synergistic with those effects that are elicited by drastic changes in satiety state (only on the left side).

This finding supports early results that indicated some kind of functional redundancy between SCN lobes that apparently do not differ in terms of their circadian properties [46–48]. Since then, there is an increasing knowledge on the capability of the left and right SCN in oscillating with asynchrony evoking antiphase rhythms in so-called “split hamsters” [44, 45, 49, 50]. Therefore, the way of thinking on redundant SCN task management should be deliberated; however, the evolutionary role and ergonomic advantages of the possibility of this optional (and condition-dependent) asymmetric working are far from being understood.

## Conclusions

According to the present results, in male animals, regulation of food intake and metabolism is coordinated by the hypothalamic hemispheres in a lateralized manner. In contrast to females, this hypothalamic asymmetry does not seem to depend on the presence of gonads or reproductive state.

Beyond satiety state, in male animals, circadian events provoked strong but only slightly lateralized changes in hypothalamic metabolism. If this was the case in females as well, it would be reasonable to conclude that in females, the strong lateralization of metabolic changes related to reproductive control may easily mask the lateralization observed in the present study associated to the regulation of circadian events. Further studies are needed to test this hypothesis.

## References

[1] Harris JA, Guglielmotti V, Bentivoglio M (1996). Diencephalic asymmetries. Neurosci Biobehav Rev.

[2] Aizawa H (2013). Habenula and the asymmetric development of the vertebrate brain. Anat Sci Int.

[3] Hou G, Yang X, Yuan T-F (2013). Hippocampal asymmetry: differences in structures and functions. Neurochem Res.

[4] Gerendai I, Rotsztejn W, Marchetti B, Kordon C, Scapagnini U (1978). Unilateral ovariectomy-induced luteinizing hormone-releasing hormone content changes in the two halves of the mediobasal hypothalamus. Neurosci Lett.

[5] Nance DM, White JP, Moger WH (1983). Neurol regulation of the ovary: evidence for hypothalamic asymmetry in endocrine control. Brain Res Bull.

[6] Fukuda M, Yamanouchi K, Nakano Y, Furuya H, Arai Y (1984). Hypothalamic laterality in regulating gonadotropic function: unilateral hypothalamic lesion and ovarian compensatory hypertrophy. Neurosci Lett.

[7] López E, Cruz ME, Domínguez R (1997). Asymmetrical effects of the unilateral implant of pilocarpine on the preoptic-anterior hypothalamic area on spontaneous ovulation of the adult rat. Arch Med Res.

[8] Cruz ME, Jaramillo LP, Domínguez R (1989). Asymmetric ovulatory response induced by a unilateral implant of atropine in the anterior hypothalamus of the cyclic rat. J Endocrinol.

[9] Toth I, Kiss DS, Goszleth G, Bartha T, Frenyo LV, Naftolin F, Horvath TL, Zsarnovszky A (2014). Hypothalamic sidedness in mitochondrial metabolism: new perspectives. Reprod Sci.

[10] Toth I, Kiss DS, Jocsak G, et al. Estrogen- and satiety state-dependent metabolic lateralization in the hypothalamus of female rats. Chowen JA, ed*.* PLoS One. 2015;10(9):e0137462. 10.1371/journal.pone.0137462 10.1371/journal.pone.0137462 PMC4560379 26339901

[11] Allen LG, Hodson CA, Burden HW, Lawrence IE (1983). Effect of vagotomy on postcastration gonadotropin secretion in male rats. Proc Soc Exp Biol Med.

[12] Gerendai I, Csaba Z, Csernus V (1995). Lateralized effect of right- and left-sided vagotomy on testicular steroidogenesis and serum gonadotropin levels in hemicastrated peripubertal rats. Neuroendocrinol Lett.

[13] Gerendai I, Motta M (1990). Effect of unilateral vagotomy on serum gonadotropin concentration in rats with two testes and in hemicastrates. Endocrinol Exp.

[14] Suzuki Y, Arai Y (1986). Laterality associated with sexual dimorphism in the volume of the mouse hypogastric ganglion. Exp Neurol.

[15] Gy B, Tsibezov VV, Sjutkin EA, Veselova SP, Novikov ID, Krivosheev OG (1984). Lateralization of LH-RH in rat hypothalamus. Brain Res.

[16] Nance DM, Moger WH (1982). Ipsilateral hypothalamic deafferentation blocks the increase in serum FSH following hemi-castration. Brain Res Bull.

[17] Inase Y, Machida T (1992). Differential effects of right-sided and left-sided orchidectomy on lateral asymmetry of LHRH cells in the mouse brain. Brain Res.

[18] Cone RD, Cowley MA, Butler AA, Fan W, Marks DL, Low MJ (2001). The arcuate nucleus as a conduit for diverse signals relevant to energy homeostasis. Int J Obes Relat Metab Disord.

[19] Palkovits M (2003). Hypothalamic regulation of food intake. Ideggyogy Sz.

[20] Asarian L, Geary N (2006). Modulation of appetite by gonadal steroid hormones. Philos Trans R Soc B Biol Sci.

[21] Sohn EH, Wolden-Hanson T, Matsumoto AM (2002). Testosterone (T)-induced changes in arcuate nucleus cocaine-amphetamine-regulated transcript and NPY mRNA are attenuated in old compared to young male Brown Norway rats: contribution of T to age-related changes in cocaine-amphetamine-regulated transcript. Endocrinology..

[22] Anukulkitch C, Rao A, Dunshea FR, Blache D, Lincoln GA, Clarke IJ (2006). Influence of photoperiod and gonadal status on food intake, adiposity, and gene expression of hypothalamic appetite regulators in a seasonal mammal. AJP Regul Integr Comp Physiol.

[23] Williams KW, Elmquist JK (2012). From neuroanatomy to behavior: central integration of peripheral signals regulating feeding behavior. Nat Neurosci.

[24] Huang W, Ramsey KM, Marcheva B, Bass J (2011). Circadian rhythms, sleep, and metabolism. J Clin Invest.

[25] Vanetsian GL, Pavlova IV (2004). Functional asymmetry of the frontal cortex and lateral hypothalamus of cats during an operant food-related conditioned reflex. Neurosci Behav Physiol.

[26] Swann JM, Turek FW (1985). Multiple circadian oscillators regulate the timing of behavioral and endocrine rhythms in female golden hamsters. Science..

[27] Illius AW, Tolkamp BJ, Yearsley J (2002). The evolution of the control of food intake. Proc Nutr Soc.

[28] Laughlin SB, de Ruyter van Steveninck RR, Anderson JC (1998). The metabolic cost of neural information. Nat Neurosci.

[29] Kann O, Schuchmann S, Buchheim K, Heinemann U (2003). Coupling of neuronal activity and mitochondrial metabolism as revealed by NAD(P)H fluorescence signals in organotypic hippocampal slice cultures of the rat. Neuroscience..

[30] Kann O, Kovács R (2007). Mitochondria and neuronal activity. Am J Physiol Cell Physiol.

[31] Kiss DS, Toth I, Jocsak G, Sterczer A, Bartha T, Frenyo LV, Zsarnovszky A (2016). Preparation of purified perikaryal and synaptosomal mitochondrial fractions from relatively small hypothalamic brain samples. MethodsX..

[32] Brand MD, Nicholls DG (2011). Assessing mitochondrial dysfunction in cells. Biochem J.

[33] Mizunuma H, DePalatis LR, McCann SM (1983). Effect of unilateral orchidectomy on plasma FSH concentration: evidence for a direct neural connection between testes and CNS. Neuroendocrinology..

[34] von Ziegler NI, Lichtensteiger W (1992). Asymmetry of brain aromatase activity: region- and sex-specific developmental patterns. Neuroendocrinology..

[35] Naftolin F, Garcia-Segura LM, Horvath TL, Zsarnovszky A, Demir N, Fadiel A, Leranth C, Vondracek-Klepper S, Lewis C, Chang A, Parducz A (2007). Estrogen-induced hypothalamic synaptic plasticity and pituitary sensitization in the control of the estrogen-induced gonadotrophin surge. Reprod Sci.

[36] Nasrallah CM, Horvath TL (2014). Mitochondrial dynamics in the central regulation of metabolism. Nat Rev Endocrinol.

[37] Mittleman G, Fray PJ, Valenstein ES (1985). Asymmetry in the effects of unilateral 6-OHDA lesions on eating and drinking evoked by hypothalamic stimulation. Behav Brain Res.

[38] Grundmann SJ, Pankey EA, Cook MM, Wood AL, Rollins BL, King BM (2005). Combination unilateral amygdaloid and ventromedial hypothalamic lesions: evidence for a feeding pathway. Am J Physiol Regul Integr Comp Physiol.

[39] Pavlova IV, Mats VN (1996). The functional asymmetry of the rabbit lateral hypothalamus during food motivation. Zh Vyssh Nerv Deiat Im I P Pavlova.

[40] Regard M, Landis T (1997). Gourmand syndrome: eating passion associated with right anterior lesions. Neurology..

[41] Craig AD (2003). Interoception: the sense of the physiological condition of the body. Curr Opin Neurobiol.

[42] Uher R, Treasure J (2005). Brain lesions and eating disorders. J Neurol Neurosurg Psychiatry.

[43] Craig ADB (2005). Forebrain emotional asymmetry: a neuroanatomical basis?. Trends Cogn Sci.

[44] de la Iglesia HO, Meyer J, Schwartz WJ (2003). Lateralization of circadian pacemaker output: activation of left- and right-sided luteinizing hormone-releasing hormone neurons involves a neural rather than a humoral pathway. J Neurosci.

[45] de la Iglesia HO, Meyer J, Carpino A, Schwartz WJ (2000). Antiphase oscillation of the left and right suprachiasmatic nuclei. Science..

[46] Donaldson JA, Stephan FK (1982). Entrainment of circadian rhythms: retinofugal pathways and unilateral suprachiasmatic nucleus lesions. Physiol Behav.

[47] Pickard GE, Turek FW (1983). The suprachiasmatic nuclei: two circadian clocks?. Brain Res.

[48] Evans JA, Elliott JA, Gorman MR (2010). Dynamic interactions between coupled oscillators within the hamster circadian pacemaker. Behav Neurosci.

[49] Gibson EM, Humber SA, Jain S, Williams WP 3rd, Zhao S, Bentley GE, Tsutsui K, Kriegsfeld LJ (2008). Alterations in RFamide-related peptide expression are coordinated with the preovulatory luteinizing hormone surge. Endocrinology..

[50] Tavakoli-Nezhad M, Schwartz WJ (2005). C-Fos expression in the brains of behaviorally “split” hamsters in constant light: calling attention to a dorsolateral region of the suprachiasmatic nucleus and the medial division of the lateral habenula. J Biol Rhythm.

